# Prophylactic alpha blockers fail to prevent postoperative urinary retention following orthopaedic procedures: evidence from a meta-analysis and trial sequential analysis of comparative studies

**DOI:** 10.3389/fphar.2023.1214349

**Published:** 2023-08-25

**Authors:** Lianliang Shan, Ping Sun, Wenping Zhang, Xuelian Zheng, Hua Li, Songling Wang

**Affiliations:** ^1^ Department of Hand Surgery/Foot and Ankle Surgery, Shengli Oilfield Central Hospital, Dongying, Shandong, China; ^2^ Dongying Vocational Institute, Dongying, Shandong, China; ^3^ Department of Bone/Joint Surgery and Sports Medicine Center, The First Affiliated Hospital of Jinan University, Guangzhou, Guangdong, China

**Keywords:** orthopaedic procedures, prophylactic alpha blockers, postoperative urinary retention, systematic review and meta-analysis, trial sequential analysis

## Abstract

**Objective:** The present systematic review and meta-analysis aimed to estimate the prophylactic effect of alpha blockers against postoperative urinary retention (POUR) in orthopaedic patients.

**Methods:** PubMed, Embase, Web of Science and Cochrane Library databases were searched between 1 January 1990 and 1 March 2023. The studies reporting the preventive efficacy of alpha blockers on POUR after orthopaedic procedures were identified. The pooled rates of POUR in the Intervention group (patients receiving alpha blockers) and the Control group (patients not receiving alpha blockers) were estimated and compared. The risk ratios (RRs) were calculated using the random-effects model. Subgroup analysis was performed based on surgical type. Trial sequential analysis (TSA) was conducted to confirm the robustness of pooled results.

**Results:** Seven studies containing 1,607 patients were identified. The rates of POUR were similar between the two groups (Intervention group: 126/748 [16.8%] VS. Control group: 168/859 [19.6%]; RR = 0.75; 95% confidence interval [CI] 0.51 to 1.09; *p* = 0.130; Heterogeneity: I^2^ = 67.1%; *p* = 0.006). No significant difference in the incidence of POUR was observed in either the Arthroplasty subgroup or Spine surgery subgroup. The result of TSA suggested that the total sample size of the existing evidence might be insufficient to draw conclusive results. Administrating alpha blockers was associated with a higher risk of complications (88/651 [13.5%] VS. 56/766 [7.3%]; RR = 1.73; 95% CI 1.27 to 2.37; *p* = 0.0005; Heterogeneity: I^2^ = 0%; *p* = 0.69).

**Conclusion:** Prophylactic alpha blockers do not reduce the risk of POUR in orthopaedic procedures, and administrating these drugs was associated with a higher risk of complications.

**Systematic Review Registration:** https://www.crd.york.ac.uk/PROSPERO/display_record.php?RecordID=409388.

## 1 Introduction

Postoperative urinary retention (POUR) is generally defined as the difficulty in completing micturition in the presence of a full bladder after surgery (Darrah et al., 2009). The incidence of POUR ranges from 3% to 70% depending on the type of surgery (Baldini et al., 2009). This variability is also evident in the field of orthopaedic surgery. Törn et al. documented a POUR prevalence of 75% among patients undergoing total joint arthroplasty (Törn et al., 1994). Boulis et al. reviewed the records of 503 patients following spine surgery and observed a POUR incidence of up to 38% (Boulis et al., 2001). POUR is distressing and painful for patients and can affect postoperative outcomes and patient satisfaction. Although catheterization is often deemed an effective solution for POUR, the intervention itself is usually uncomfortable and can introduce the risk of increased hospital expenses and delayed discharge (Foxman, 2003; Parker et al., 2017).

POUR and the use of urinary catheterization may increase the predisposition to urinary tract infections (UTIs), which is a risk factor for the development of implant-related infection, a catastrophic complication in orthopaedic procedures (Yoon and King, 2020; Pertsch et al., 2021; Wang et al., 2021). Hence, there has been a growing endorsement of pharmacologic interventions aimed at reducing POUR occurrence in orthopaedic procedures.

Alpha blockers have been approved for the treatment of hypertension, benign prostatic hyperplasia and neurogenic bladder (Khoury and Kaplan, 1991; Kakizaki et al., 2003). Among these, alpha-1 antagonists, including prazosin, demonstrate the ability to induce vasodilation and promote urinary smooth muscle relaxation. Tamsulosin is a kind of super-selective alpha-1A antagonist with greater affinity for the urinary system, which mainly inhibits urinary smooth muscle contraction and facilitates voiding. The off-label uses of alpha blockers in POUR have also been explored in diverse surgical populations. Chapman et al. reported that tamsulosin was effective in preventing POUR following female pelvic reconstructive surgery (Lose and Lindholm, 1985; Chapman et al., 2021). Similarly, Gönüllü et al. found that prophylactic administration of prazosin could reduce the incidence of POUR and catheterization following herniorrhaphy (Gönüllü et al., 1999).

Nevertheless, the assessment of alpha blockers’ effects on POUR in orthopaedic procedures remains limited and has yielded conflicting results. Choi et al. conducted a randomized controlled trial (RCT) investigating prophylactic tamsulosin in 95 patients undergoing total joint arthroplasty and found that the use of tamsulosin could curtailthe risk of POUR by two-thirds (*p* = 0.044) (Choi et al., 2021). In contrast, a controversial outcome was observed in the RCT by Schubert et al., wherein the patients receiving tamsulosin exhibited a comparable incidence of POUR with those not receiving the drug after arthroplasty (Tamsulosin: 28.1% VS. Control: 35.8%, *p* = 0.345) (Schubert et al., 2019). Basheer et al. also detected limited effectiveness of perioperative tamsulosin in mitigating the rate of POUR in spine surgery (Basheer et al., 2017). Given these uncertain findings, determining the prophylactic efficacy of alpha blockers against POUR in orthopaedic procedures remains a subject of interest and debate.

Therefore, the objective of this systematic review and meta-analysis is (Darrah et al., 2009): to identify whether prophylactic alpha blockers can diminish the risk of POUR, and (Baldini et al., 2009) to ascertain the presence of any potential complications associated with the administration of alpha blockers.

## 2 Methods

This systematic review and meta-analysis was conducted following the Preferred Reporting Items for Systematic reviews and Meta-Analyses (PRISMA) Statement protocol (CRD42023409388) (Shamseer et al., 2015; Cumpston et al., 2019).

### 2.1 Search strategy and eligibility

PubMed, Embase, Web of Science and Cochrane Library databases were searched between 1st January 1990 and 1st March 2023. We developed search strategies for each database according to the principle of PICOS (Population, Intervention, Comparison, Outcome, Study design), and the references of the identified studies were checked for potential eligibility (Supplementary Table S1). Specifically, the search strategy for PubMed was: (alpha blocker OR alpha blockade OR alpha antagonist OR prazosin OR phenoxybenzamine OR doxazosin OR terazosin OR alfuzosin OR silodosin OR tamsulosin) AND (urinary retention OR voiding difficulty). The keywords regarding orthopaedic procedures were deliberately omitted during the initial search to avoid the exclusion of potentially relevant studies, given the diversity of orthopaedic procedures.

Eligible studies were identified using the following inclusion criteria: 1) patients following orthopaedic surgery as the interested population; 2) alpha blocker as the intervention; 3) placebo or no treatment as the comparison; 4) incidence of POUR as the primary outcome; 5) with comparative study design.

We excluded non-English language reports, *in vitro* studies, case reports, brief reports, conference abstracts/posters or reviews. After removing duplicates, titles and abstracts were reviewed to identify eligible papers. Full texts were assessed to determine the final list of publications eligible for inclusion in the study.

### 2.2 Data extraction

After the final list of included studies was set, we extracted the following information: year of publication, patient age, definition of POUR, operative information, administration of drugs and study design. The primary outcome of interest was the incidence of POUR. Complications were extracted as secondary outcomes.

### 2.3 Assessment of quality and bias

The quality of the included studies was assessed independently by two authors. The modified Jadad Scale was employed for RCTs (Moher et al., 1996), and the Newcastle-Ottawa Scale for cohort studies (Stang, 2010). The publication bias was estimated using the funnel plot and Harbord’s test (Harbord et al., 2006). In case of disagreement, a third senior doctor was consulted.

### 2.4 Statistical analysis

Statistical analysis was performed using RevMan software (version 5.3, Cochrane Collaboration, Oxford, the United Kingdom), R software (version 4.1.3, R Foundation for Statistical Computing, Vienna, Austria) and Trial Sequential Analysis (TSA) software (version 0.9.5.10 beta, Copenhagen Trial Unit, Copenhagen, Denmark), with *p* < 0.05 as the threshold for statistical significance. Heterogeneity among the studies was evaluated through the I^2^ statistic and Q test. Generally, a fixed-effects model is employed to calculate pooled results if I^2^ is less than 50% and the *p*-value for the Q test exceeds 0.05. Conversely, a random-effects is employed if I^2^ exceeds 50% or the *p*-value for the Q test is below 0.05. In this meta-analysis, we preferred the random-effects model to the fixed-effects model due to the inclusion of various alpha blockers and diverse dosing regimens. We postulated that the effectiveness of these interventions might exhibit variability across studies. Thus, a random-effects model was prespecified (Borenstein et al., 2010; Nikolakopoulou et al., 2014). The pooled rate of POUR or complications was calculated using the Mantel-Haenszel (M-H) method with a 95% confidence interval (CI). To ensure the robustness of the primary outcome of POUR, we evaluated the risk of false positives or false negatives using a *post hoc* TSA. Sequential boundaries were set according to a type I error of 5% and a power of 80%. The required information size (RIS) was calculated to determine whether the sample size was adequate to reach a reliable conclusion. The cumulative curve of the Z score was plotted. Sensitivity analysis was conducted using the leave-one-out analysis. Subgroup analysis was performed stratified by the type of operation.

## 3 Results

### 3.1 Overview of search results

**FIGURE 1:**
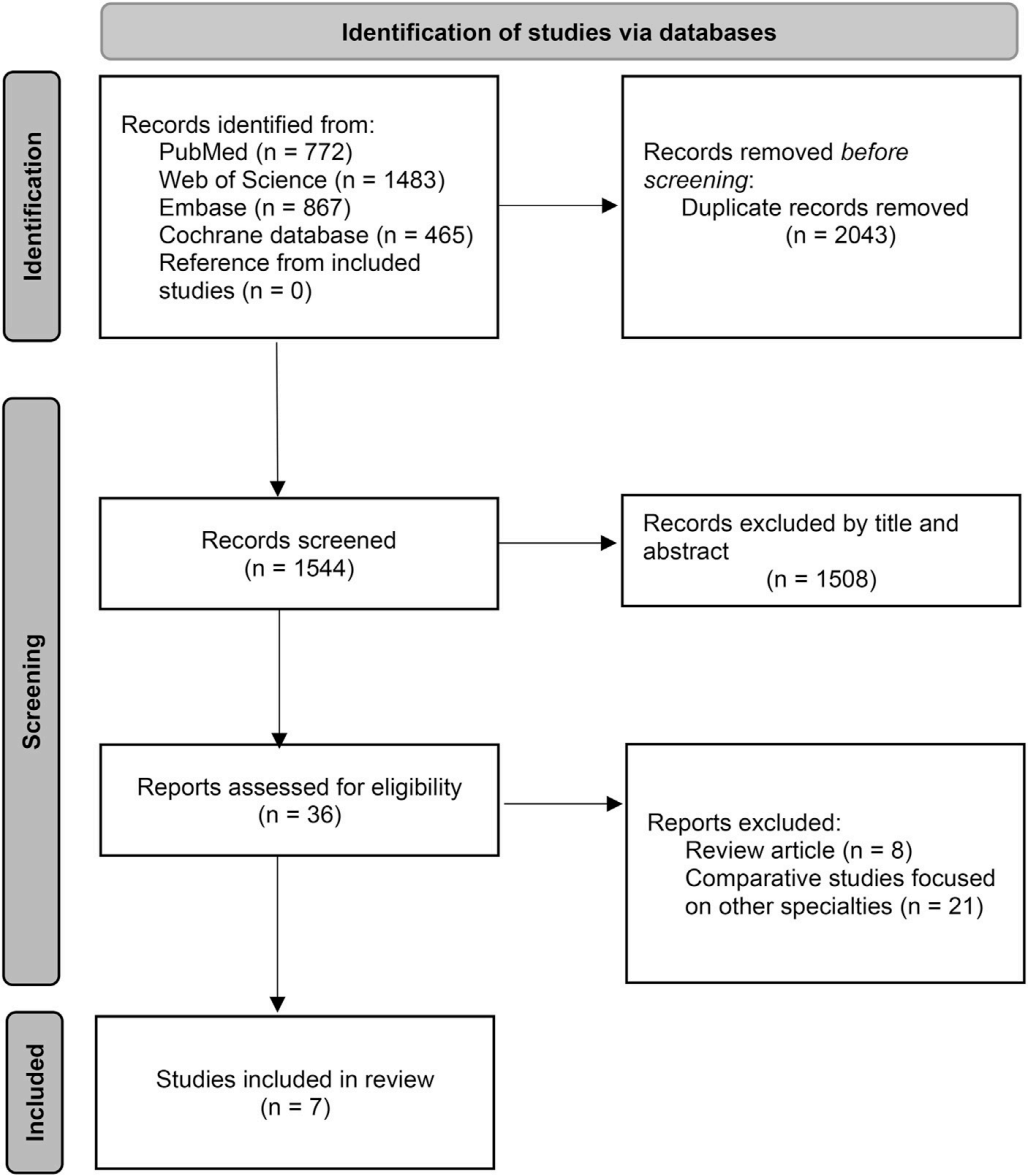
Flowchart of PRISMA.

A total of 3,587 studies were identified at the initial search. After removing duplicates, 1,544 records were screened by titles and abstracts. Of these, 36 papers were assessed for eligibility by reading the full texts. Finally, seven studies (six RCTs and one retrospective cohort study) were included in the analysis (Figure 1) (Petersen et al., 1991; Basheer et al., 2017; Schubert et al., 2019; Schubert et al., 2020; Choi et al., 2021; Ding et al., 2022; Rughani et al., 2022). Among these studies, five focused on patients undergoing arthroplasty, while two investigated cases undergoing spine surgery. Six out of the seven studies reported the proportion of patients with a history of benign prostatic hyperplasia or urinary retention, with the cohort study exhibiting the highest proportion in this regard. Tamsulosin was the most commonly used drug. The details of the included studies were summarized in Table 1.

**TABLE 1:** Characteristics of included studies.

Authors	Year	Study design	Operative type	Reported outcomes	Definition of POUR	Interventions (I/C)
Petersen et al.	1991	RCT	TJA	POUR, complication	Inability to void with symptoms	Prazosin 2 mg every 12 h preoperatively and postoperatively throughout hospitalization/No treatment
Basheer et al.	2017	RCT	Spine surgery	POUR	PVR >250 mL	Two doses: Tamsulosin 0.4 mg; 48 h prior to surgery and the night before surgery/Placebo
Schubert et al.	2019	RCT	TJA	POUR, complication	PVR >200 mL; or urine volume retention >200 mL with inability to void within 6 h after indwelling urinary catheter removal; or urine volume retention <200 mL with symptoms and inability to void	Daily dose: Tamsulosin 0.4 mg; 5 days prior to surgery, the morning of surgery, and on the first postoperative day/Placebo
Schubert et al.	2020	Retrospective cohort study	TJA	POUR, complication	Records of intermittent straight catheterization	Tamsulosin, terazosin, doxazosin, alfuzosin, silodosin and prazosin/No treatment
Choi et al.	2021	RCT	TJA	POUR	PVR >400 mL	Daily doses: Tamsulosin 0.2 mg; 3 days after surgery/Placebo
Rughani et al.	2022	RCT	Spine surgery	POUR, complication	Urine volume retention >300 mL	Daily dose: Tamsulosin 0.4 mg; 5 days prior to surgery and 2 days after surgery/Placebo
Ding et al.	2023	RCT`	TJA	POUR, complication	Inability to void with symptoms	Doxazosin 4 mg once 2 h prior to surgery/Placebo

POUR, postoperative urinary retention; I/C, Intervention group/Control group; RCT, randomized control trial; TJA, total joint arthroplasty; PVR, post-void residual volume.

### 3.2 Assessment of quality and bias

**FIGURE 2:**
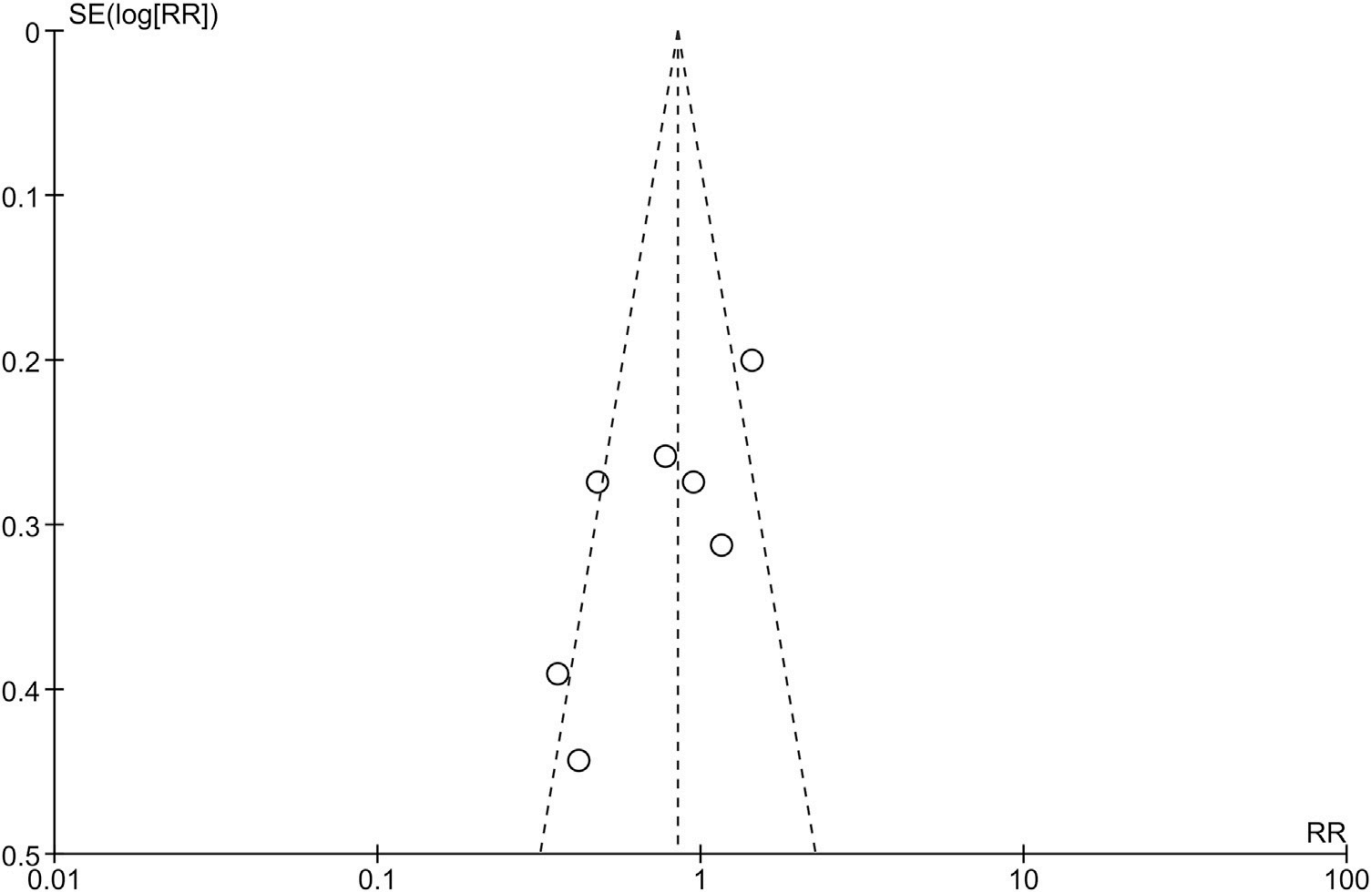
Funnel plot for publication bias.

All RCTs had a Jadad score higher than four, indicating that they were of high quality. The Newcastle-Ottawa rank also revealed the high quality of the cohort study. The details of the assessment were summarized in Table 2. The funnel plot did not show the concerns of possible publication bias (Figure 2), which was also consistent with the formal test (Harbord’s test, *p* = 0.145).

**TABLE 2:** Details of quality assessment.

Research										
RCT/Jadad Score	Randomization				Concealment	Blinded	Withdraw or drop-out			Total
Schubert et al. (2019)	2				2	2	1			7
Basheer et al.	2				1	2	0			5
Rughani et al.	2				2	2	1			7
Choi et al.	2				1	2	1			6
Petersen et al.	1				1	2	1			5
Ding et al.	2				2	2	1			7

Non-RCT/Newcastle-Ottawa Scale	Selection				Comparability		Outcome			Total
	1	2	3	4	+		1	2	3	
Schubert et al. (2020)	+	+	+	+	+		+	+	+	8+

### 3.3 Primary outcome

**FIGURE 3:**
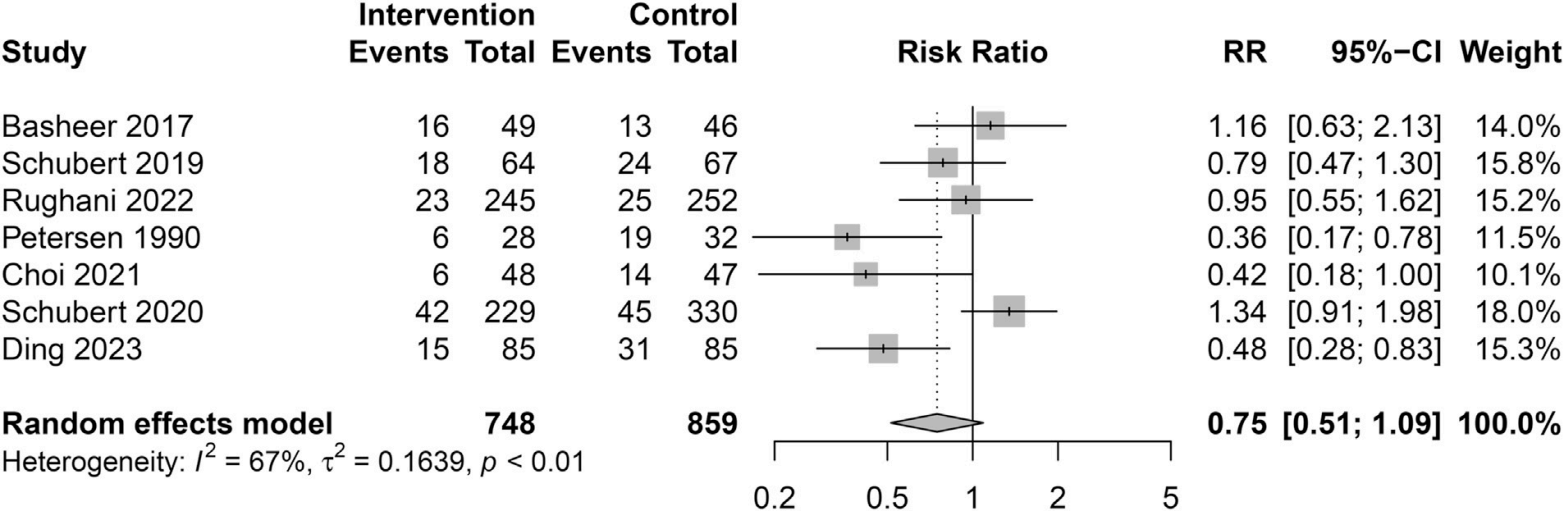
Forest plot for the incidence of POUR.

**FIGURE 4:**
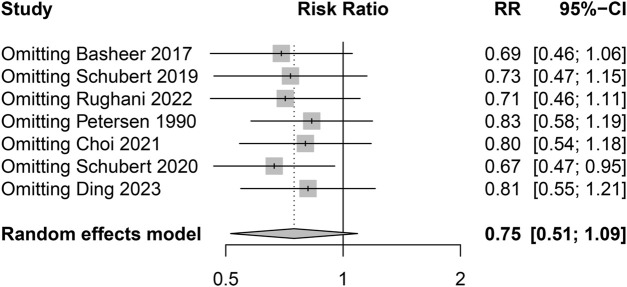
The sensitivity analysis using the leave-one-out analysis.

A total of 748 patients (Male: 95.6% VS. Female: 4.4%) received alpha blockers (Intervention group), while 859 patients (Male: 96.2% VS. Female: 3.8%) received a placebo or no intervention (Control group). Overall, there were 294 episodes of POUR in 1,607 patients (18.3%). The pooled analysis showed no significant difference in the incidence of POUR between the two groups with moderate heterogeneity (Intervention group: 126/748 [16.8%] VS. Control group: 168/859 [19.6%]; RR = 0.75; 95% CI 0.51 to 1.09; *p* = 0.130; Heterogeneity: I^2^ = 67.1%; *p* = 0.006) (Table 3; Figure 3). The sensitivity analysis using the leave-one-out analysis revealed that the exclusion of the study by Schubert et al. (2020) (Schubert et al., 2020), representing the only retrospective cohort study in this meta-analysis, demonstrated a statistically significant impact on the pooled results (Figure 4).

**TABLE 3:** Major findings of included papers.

Authors	Sample size (I/C) (n)	Gender (M/F)	Mean age (y)	History of BPH or UR	POUR (I/C) (n)	Complications (I/C)
Petersen et al.	28: 14 TKA, 12 THA, 2 revision/32: 13 TKA, 17 THA, 2 revision	All male	65.4	NA	6/19	3 UTI/3 UTI
Basheer et al.	49: 23 C-spine surgery, 2 T-spine surgery, 24 L-spine surgery/46: 18 C-spine surgery, 2 T-spine surgery, 26 L-spine surgery	All male	57.4	13.7%	16/13	NA
Schubert et al. (2019)	64: 21 TKA, 43 THA/67: 20 TKA, 47 THA	All male	61.0	0	18/24	24: 2 UTI, 2 floppy iris syndrome, 1 dizziness, 3 syncope, 2 constipation, 8 increased pain, 1 viral infection, 1 pruritus, 1 mental disorder, 1 shortness of breath, 1 fatigue, 1 GI bleed/11: 0 UTI, 1 hypotension, 2 dizziness, 1 constipation, 2 increased pain, 1 wound dehiscence, 2 SSI, 1 GI bleed, 1 gout
Schubert et al. (2020)	229: 113 TKA, 1 UKA, 106 THA, 9 revision/330: 146 TKA, 184 THA	All male	66.6	44.4%	42/45	17 UTI/12 UTI
Choi et al.	48: 14 TKA, 34 THA/47: 16 TKA, 31 THA	29/66	68.3	0	6/14	NA
Rughani et al.	245: 68 C-spine surgery, 2 T-spine surgery, 175 L-spine surgery/252: 53 C-spine surgery, 1 T-spine surgery, 198 L-spine surgery	All male	63.6	7.6%	23/25	25: 0 UTI/15: 1 UTI.
Ding et al.	85: 17 TKA, 68 THA/85: 17 TKA, 68 THA	All male	54.4	10.0%	15/31	19: 1 UTI, 3 hypotension, 2 dizziness, 2 nausea, 2 fatigue, 7 delayed healing, 1 transfusion, 1 neurovascular event/15: 2 UTI, 1 hypotension, 3 nausea, 2 fatigue, 1 pruritis, 5 delayed healing, 1 transfusion

I/C, Intervention group/Control group; n, number; M/F, Male/Female; BPH, benign prostatic hyperplasia; UR, urinary retention; POUR, postoperative urinary retention; TKA, total knee arthroplasty; THA, total hip arthroplasty; UKA, unicompartmental knee arthroplasty; UTI, urinary tract infection; GI, gastrointestinal; SSI, surgical site infection; NA, not applicable.

**FIGURE 5:**
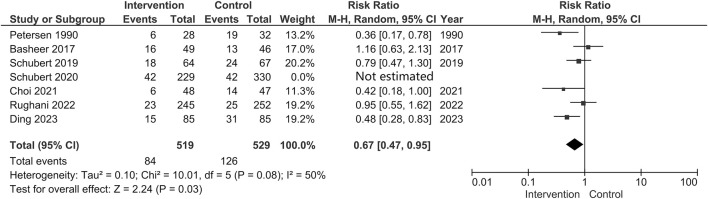
Forest plot for the incidence of POUR after omitting the only cohort study.

**FIGURE 6:**
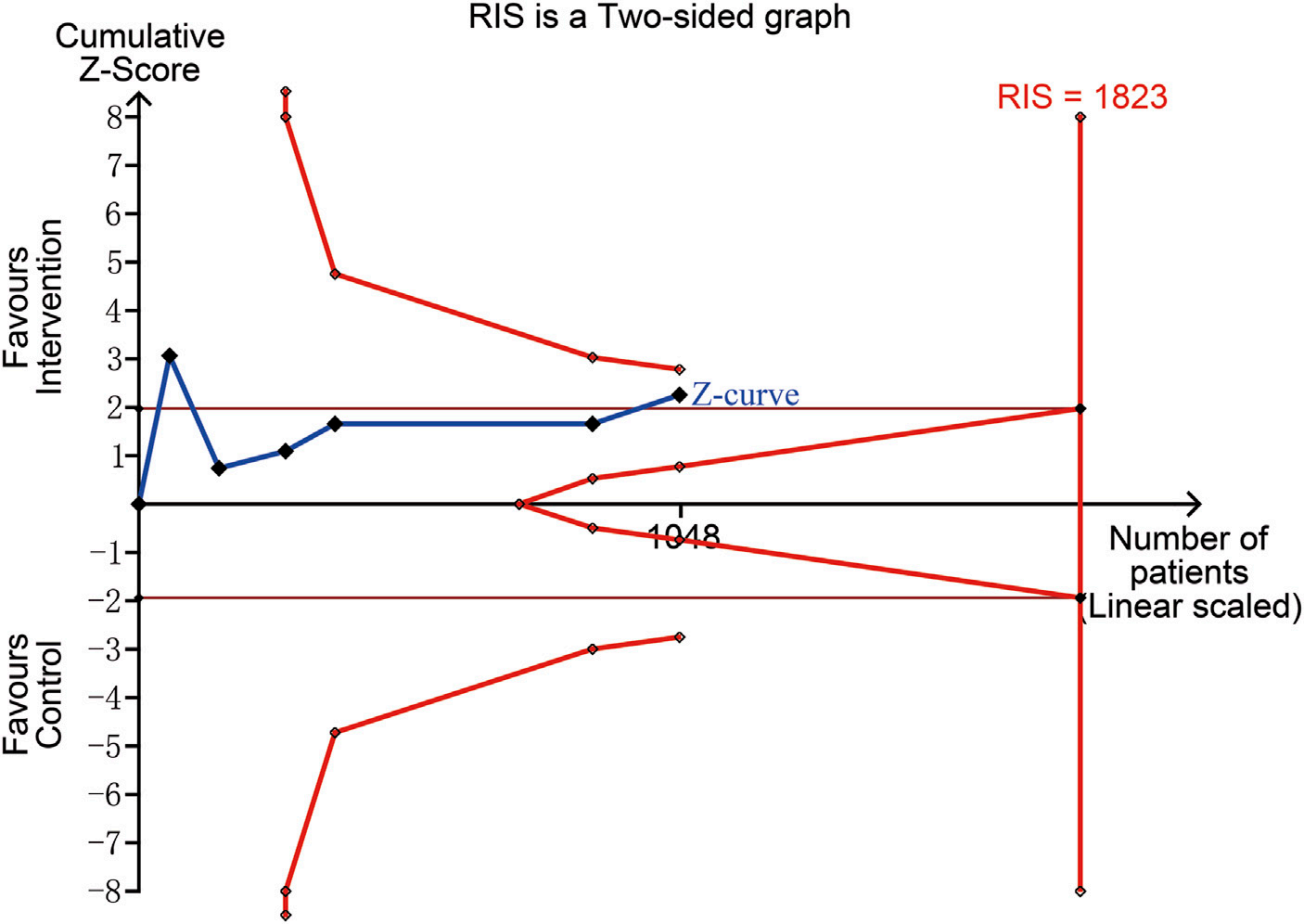
Trial sequential analysis for the incidence of POUR. The cumulative curve of the Z score (blue line) crossed naïve boundary (parallel brown line), which reflected that the difference of the incidence of POUR between the two groups were statistically significant. However, the curve did not cross the upper sequential boundary (red curved line) nor reach the required information size (RIS) (red longitudinal line), which indicated that the pooled results failed to support the benefit of alpha blockers due to the limited sample size.

Considering the level of evidence of each study, we subsequently analyzed the data only from RCTs. This analysis reflected that the pooled incidence of POUR was lower in the Intervention group than that in the Control group (Intervention group: 84/519 [16.2%] VS. Control group: 126/529 [23.8%]; RR = 0.67; 95% CI 0.47 to 0.95; *p* = 0.03; Heterogeneity: I^2^ = 50%; *p* = 0.08) (Figure 5). However, the *post hoc* TSA showed that the cumulative curve of the Z score did not cross the sequential boundary nor the RIS, suggesting that the total sample size of the existing evidence may be insufficient to draw conclusive results (Figure 6).

### 3.4 Subgroup analysis of primary outcome

The included studies were stratified into two subgroups: the Arthroplasty subgroup and the Spine surgery subgroup. In the Arthroplasty subgroup, the incidence of POUR was 19.2% in the Intervention group and 23.2% in the Control group, respectively. Two studies conducted a comparative assessment of knee and hip arthroplasty. In the study by Petersen et al., the incidence of POUR was comparable (Knee arthroplasty: 41% VS. Hip arthroplasty: 48%) (Petersen et al., 1991). Choi et al. reported that hip arthroplasty was not a risk factor for POUR as compared with knee arthroplasty (OR = 0.821, *p* = 0.711) (Choi et al., 2021).

In the Spine surgery subgroup, POUR was documented at a rate of 13.3% in the Intervention group and 12.8% in the Control group, respectively. The main surgical level was the lumbar region. The relationship between the incidence of POUR and the surgical levels remained unexplored in the two studies (Basheer et al., 2017; Rughani et al., 2022).

**FIGURE 7:**
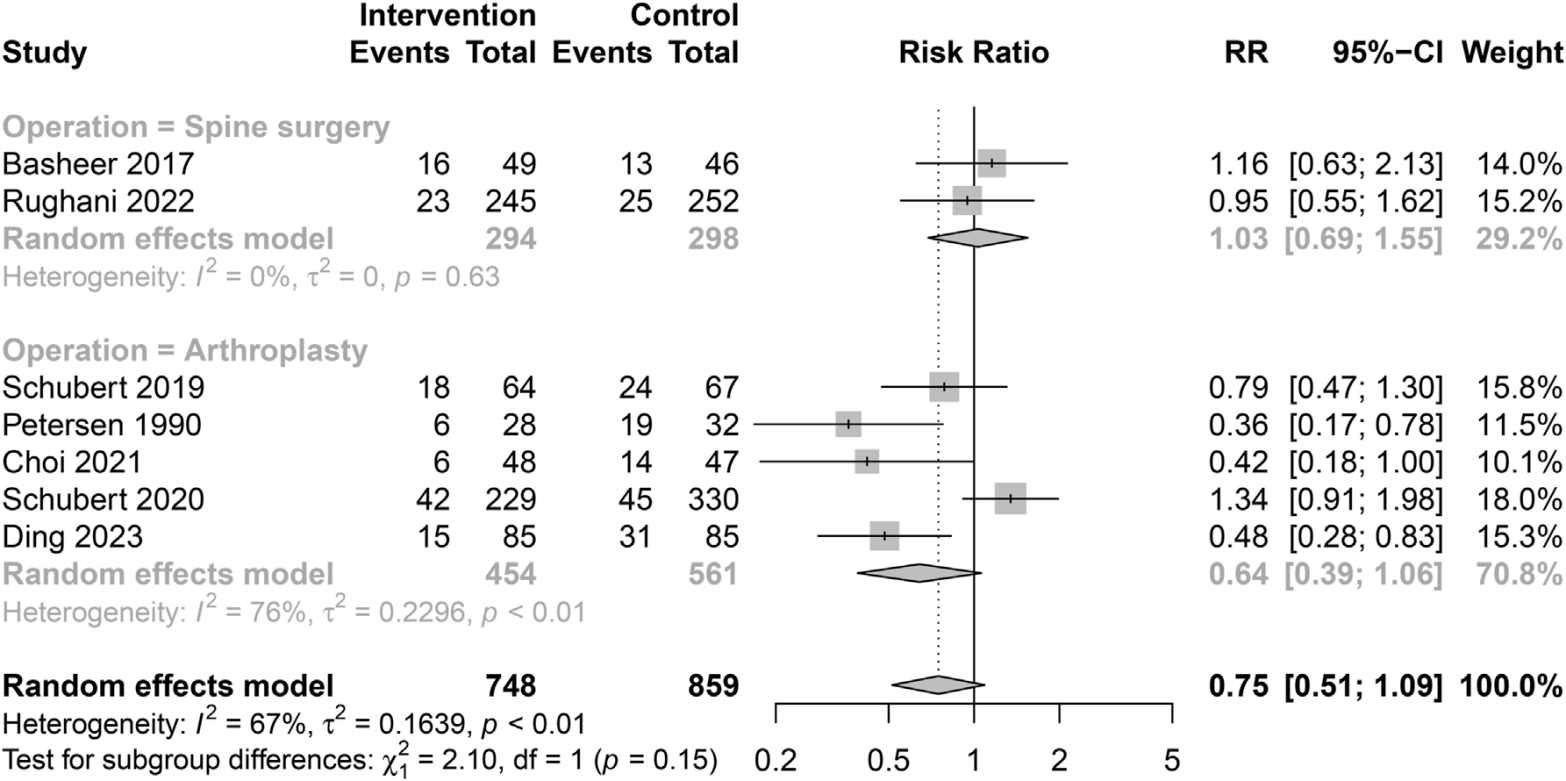
Subgroup analysis of the incidence of POUR based on the surgical type.

Prophylactic administration of alpha blockers did not show a significant protective effect in either subgroup (Arthroplasty subgroup: RR = 0.64, 95% CI 0.39 to 1.06; Spine surgery subgroup: RR = 1.03, 95% CI 0.69–1.55). Little heterogeneity was observed between the two subgroups (*p* = 0.15) (Figure 7).

### 3.5 Secondary outcomes

**FIGURE 8:**
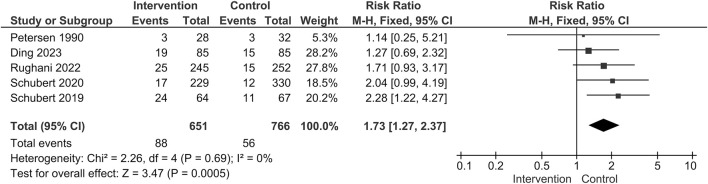
Forest plot for the incidence of complications.

**FIGURE 9:**
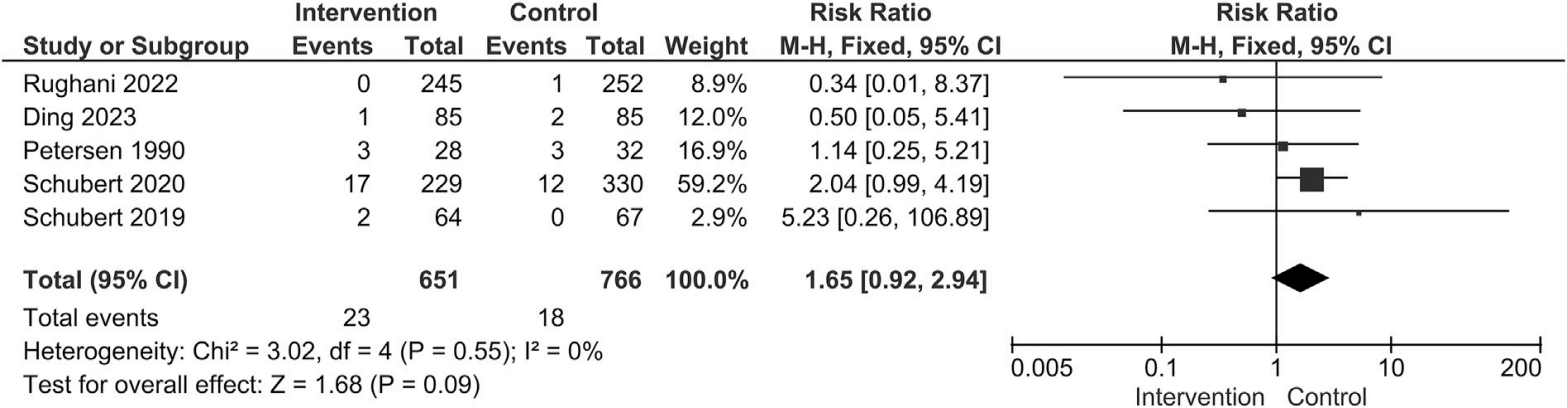
Forest plot for the incidence of UTI.

Five studies reported the data of complications and the pooled analysis showed that the administration of alpha blockers would introduce a higher risk of complications (88/651 [13.5%] VS. 56/766 [7.3%]; RR = 1.73; 95% CI 1.27 to 2.37; *p* = 0.0005; Heterogeneity: I^2^ = 0%; *p* = 0.69) (Figure 8) (Petersen et al., 1991; Schubert et al., 2019; Schubert et al., 2020; Ding et al., 2022; Rughani et al., 2022). UTI was the most commonly described and concerned complication. The rate of UTI was similar between the two groups (23/651 [3.5%] VS. 18/766 [2.3%]; RR = 1.65; 95% CI 0.92 to 2.94; *p* = 0.09; Heterogeneity: I^2^ = 0; *p* = 0.55) (Figure 9).

## 4 Discussion

Based on available evidence, we failed to identify a significant efficacy of prophylactic alpha blockers in reducing the risk of POUR after orthopaedic procedures. The pooled analysis of seven studies demonstrated that the incidence of POUR was comparable between the Intervention group and Control group (16.8% VS. 19.6%, respectively). Although the pooled results of six RCTs represented the highest level of evidence, suggesting a statistically significant 33% reduction in the risk of POUR (RR = 0.67; 95% CI 0.47–0.95) associated with alpha blockers, this finding must be approached with caution due to the relatively limited sample size. In addition, we found that the prophylactic alpha blockers might potentially increase the risk of complications.

### 4.1 Incidence of POUR

Our research holds clinical importance as it seeks to investigate the efficacy of pharmacologic interventions against POUR in orthopaedic patients. However, our findings showed a lack of statistically significant difference in the incidence of POUR when comparing cases with or without the administration of alpha blockers. These results differed from previous studies and meta-analyses in other clinical disciplines. Ghuman et al. conducted a meta-analysis of 15 RCTs in general surgery and urologic surgery and found that the prophylactic use of alpha blockers could yield a 17.1% absolute reduction in POUR risk (Ghuman et al., 2018). Another meta-analysis encompassing five RCTs revealed a reduction of 20.6% in the risk of POUR among patients after hernia repair following the administration of alpha blockers (Clancy et al., 2018). The discrepancy between our results and prior findings may be attributed to multiple factors. First, orthopaedic patients have an elevated inherent susceptibility to POUR owing to multiple factors, such as advanced age demographics, frequent use of neuraxial anaesthesia, and routine employment of indwelling urinary catheters (Schubert et al., 2019; Leitner et al., 2021). As a result, the perceived effectiveness of alpha blockers might not manifest to the same extent as witnessed in other disciplines. Second, orthopaedic procedures are typically major surgeries (Hoogeboom et al., 2014; Dietz et al., 2019), which pose a challenge for cautious perioperative fluid management (Heming et al., 2020). Arthroplasty and spine surgery often entails substantial intraoperative fluid administration (exceeding 1,000 mL), which is also linked to a potential risk of POUR (Lee et al., 2017; Aiyer et al., 2018; Halawi et al., 2019; Ongaigui et al., 2020). Third, our *post hoc* TSA to evaluate the robustness of our findings provided a more conservative estimation. It could be inferred that our aggregated results of RCTs might be false positive due to a relatively small sample size. Thus, more high-level evidence is required to determine the true efficacy of alpha blockers against POUR in orthopaedic patients.

### 4.2 Risk factors of POUR for orthopaedic procedures

Only two kinds of orthopaedic procedures, arthroplasty and spine surgery, were included in our review despite a thorough investigation across four main databases. In terms of lower-limb arthroplasty, our subgroup data indicated that the efficacy of alpha blockers seemed to be consistent between knee and hip procedures. However, it should be noted that the incidence of POUR might not be close between these two procedures. Abdul-Muhsin et al. reported that knee arthroplasty had a higher prevalence of POUR than hip arthroplasty (Abdul-Muhsin et al., 2020). As for the spine surgery, the majority of cases in our review pertained to lumbar-level interventions, which was reported to be associated with an elevated susceptibility to POUR as compared with surgeries targeting the cervical or thoracic regions (Altschul et al., 2017). Due to the limited available data, our review was unable to explore potential correlations between surgical levels and drug efficacy.

Information regarding the use of alpha blockers to prevent POUR in other orthopaedic surgeries is lacking. Several studies have identified the risk factors and preventive regimens for patients following different orthopaedic surgeries. Tobu et al. found that patients with femoral neck fractures who had dementia and/or delirium had a tenfold higher risk of developing POUR (Tobu et al., 2014). Higashikawa et al. also found that cognitive impairment in female patients with proximal hip fractures was associated with a higher likelihood of POUR (Higashikawa et al., 2019). The authors recommended necessary nursing care in activities of daily living and neurofunctional assistance to mitigate the risk. A history of benign prostatic hyperplasia or neurogenic bladder is also a well-established risk factor. A meta-analysis showed that patients with such a condition were associated with a 3.8-fold risk of developing POUR after spine surgery (Chang et al., 2021). In our meta-analysis, a strategic exclusion of patients with a history of benign prostatic hyperplasia was instituted across most RCTs to mitigate potential bias. However, this discerning protocol also attenuated the generalizability of the pooled results. Further studies are appealed to explore the strategy against POUR in patients at higher risk, such as those with benign prostatic hyperplasia or neurogenic bladder. Other risk factors including spinal/epidural anaesthesia, advanced age and excessive fluid administration should also be considered in orthopaedic surgeries (Cha et al., 2020). Santini et al. adopted the International Prostate Symptom Score to assess the risk of POUR and found that a score above 18 was strongly related to a predisposition to POUR (Santini et al., 2019). However, to our knowledge, few scoring systems for POUR in orthopaedic surgeries have been widely used with a well-validated prediction (Bracey et al., 2022).

### 4.3 Concerns about complications

Our research found that the administration of alpha blockers was related to a higher risk of complications while the incidence of UTI was similar between the two groups. Other reported complications included dizziness and vomiting, which might result from vasodilation caused by the drugs. Previous studies have also raised concerns about alpha blockers, such as the carcinogenicity of phenoxybenzamine and the cardiovascular effects of prazosin (Hasford et al., 1991; Suarez-Torres et al., 2021). In our included studies, Schubert et al. reported two cases of tamsulosin-induced floppy iris syndrome (Schubert et al., 2019). Bell et al. found that the risk of floppy iris syndrome in patients after cataract surgery was 2.3 times higher when tamsulosin was administered (Bell et al., 2009), and the authors attributed this complication to the mechanism that the alpha-1A adrenergic receptors were also present in the dilator smooth muscle of the iris, and thus tamsulosin might compromise mydriasis. Surgeons should pay attention to our assembled data and inform patients of the associated complications.

### 4.4 Limitations

We noted several limitations in our meta-analysis. First, the possibility of missing relevant studies cannot be completely avoided, which may introduce bias into our methodology. Second, one of the included studies utilized a cohort study design, which may affect the level of evidence of our pooled data. However, we performed a leave-one-out analysis and calculated the pooled results after excluding the cohort study. We used the TSA to estimate the robustness of our conclusion, which suggested that our results of RCTs might not possess full sufficiency and conclusiveness. Third, we included all the studies with multiple alpha blockers as an intervention and assumed that the efficacy of alpha blockers was similar. We used the random-effects model to synthesize the data more conservatively. However, we still noticed that the pooled results reflected a moderate degree of heterogeneity, despite performing a subgroup analysis. Fourth, the data on POUR that were pooled were extracted directly from the articles and we found that the definitions of POUR in each study were similar but not identical, which might bring quantitative bias into the data. Fifth, only two types of orthopaedic surgeries were included, which might jeopardize the generalizability of the conclusion. The distribution of surgical subtypes between the intervention and control groups might also introduce bias.

## 5 Conclusion

The current meta-analysis of existing evidence found that prophylactic alpha blockers might not reduce the risk of POUR after orthopaedic procedures, and the TSA suggested that more trials were required. Administrating these drugs could be associated with a possibly higher risk of complications.

## Data Availability

The raw data supporting the conclusion of this article will be made available by the authors, without undue reservation.
