# Supplementary material for: Prophylactic alpha blockers fail to prevent postoperative urinary retention following orthopaedic procedures: evidence from a meta-analysis and trial sequential analysis of comparative studies
Source: Front Pharmacol. 2023 Aug 25;14:1214349. doi: 10.3389/fphar.2023.1214349 (PMC10485607; doi:10.3389/fphar.2023.1214349)
Supplement: Supplementary file 1 [file Table1.DOCX]

Supplementary Material

Prophylactic alpha blockers fail to prevent postoperative urinary retention following orthopaedic procedures: Evidence from a meta-analysis and trial sequential analysis of comparative studies

**Lianliang Shan, Ping Sun, Wenping Zhang, Xuelian Zheng, Hua Li, Songling Wang**

*** Correspondence:** Songling Wang: szwk007@163.com

Supplement Table 1. Search strategy.

| Database | Strategy |
| --- | --- |
| PubMed | #1: urinary retention OR voiding difficulty  #2: alpha blocker OR alpha blockade OR alpha antagonist OR prazosin OR phenoxybenzamine OR doxazosin OR terazosin OR alfuzosin OR silodosin OR tamsulosin  #3: ("1990/01/01"[Date - Publication] : "2023/03/01"[Date - Publication])  Results: #1 AND #2 AND #3 |
| Web of Science | #1: TS=(voiding difficulty) OR TS=( urinary retention)  #2: TS=alpha blocker OR alpha blockade OR alpha antagonist OR prazosin OR phenoxybenzamine OR doxazosin OR terazosin OR alfuzosin OR silodosin OR tamsulosin  #3: DOP=(1990-01-01/2023-03-01)  Results: #1 AND #2 AND #3 |
| Embase | #1: urinary AND retention  #2: voiding AND difficulty  #3: #1 OR #2  #4: (alpha AND blocker) OR (alpha AND blockade) OR (alpha AND antagonist) OR prazosin OR phenoxybenzamine OR doxazosin OR terazosin OR alfuzosin OR silodosin OR tamsulosin  #5: [01-01-1990]/sd NOT [02-03-2023]/sd  Results: #1 AND #2 AND #3 AND #4 AND #5 |
| Cochrane Library | #1: (alpha blocker) OR (alpha blockade) OR (alpha antagonist) OR prazosin OR phenoxybenzamine OR doxazosin OR terazosin OR alfuzosin OR silodosin OR tamsulosin  #2: urinary retention  #3: voiding difficulty  #4: #2 OR #3  #5: #1 AND #4  with Cochrane Library publication date Between Jan 1990 and Mar 2023 |
